# Development and validation of a nomogram for predicting the risk of intestinal barrier dysfunction in patients after major abdominal surgery: a prospective cohort study

**DOI:** 10.3389/fmed.2025.1606443

**Published:** 2025-08-21

**Authors:** Qinghua Zou, Ruotian Wang, Yunfang Dong, Weiming Li, Guoyun Zhao, Zhaochuan Yin, Manqing Hu, Yijun Li, Qingwen Xu, Lixing Wang, Kaiwen Shi, Hongyuan Liu, Yichen Hu, Yuanpei Zhao

**Affiliations:** ^1^Department of Gastrointestinal Surgery, The Second Affiliated Hospital of Kunming Medical University, Kunming, Yunnan, China; ^2^Department of General Surgery, Yan’an Hospital of Kunming City, Kunming, Yunnan, China; ^3^Department of Hepatobiliary and Pancreatic Surgery, The Second Affiliated Hospital of Kunming Medical University, Kunming, China; ^4^Department of Orthopedics, The First People’s Hospital of Yunnan Province, Kunming, Yunnan, China

**Keywords:** intestinal barrier dysfunction, abdominal major surgery, gastrointestinal surgery, pancreaticoduodenectomy, nomogram

## Abstract

**Background:**

Intestinal barrier dysfunction (IBDF) can lead to systemic inflammatory response syndrome and multiple organ failure, severely jeopardizing patient health. Preventing the occurrence of IBDF is crucial, but effective prediction and assessment tools are currently lacking. In this study, we aimed to construct and validate a nomogram for early prediction of the risk of IBDF in patients undergoing major abdominal surgery.

**Methods:**

A total of 684 patients undergoing major abdominal surgery were prospectively included, among whom patients from the Second Affiliated Hospital of Kunming Medical University and Kunming Haikou Hospital were assigned to the training (*n* = 480) and external validation (*n* = 204) cohorts, respectively. Univariate and multivariate logistic regression analyses were performed to screen for independent predictors of IBDF. Based on these factors, the nomogram was constructed to predict IBDF occurrence. The area under the receiver operating characteristic curve (AUC), calibration plot, decision curve analysis (DCA), and clinical impact curve (CIC) were used to evaluate the predictive performance and clinical utility of the model.

**Results:**

In the training and validation cohorts, 28.3 and 26.9% of patients experienced IBDF, respectively. The multivariate logistic regression analysis showed that surgical method, operative time, blood loss, infusion volume, albumin, interleukin-6, neutrophil-to-lymphocyte ratio, and opioid use were independent predictors of IBDF. The AUC of the IBDF nomogram based on these eight variables was 0.946 (95% CI: 0.921–0.970) and 0.944 (95% CI: 0.907–0.981) in the training and validation cohorts, respectively. The calibration curves showed good consistency, and the DCA and CIC results showed that the constructed model has good clinical applicability.

**Conclusion:**

We established and validated an IBDF-nomogram for the first time to predict the risk of IBDF in patients after major abdominal surgery. This model provides a practical tool for clinicians to identify high-risk patients with IBDF in the early stage, which may have significance in guiding clinical treatment decisions.

## Introduction

The intestine is referred to as the “central organ” of the body’s trauma stress and is also the engine of multiple organ failure in critically ill patients (1, 2). Under normal circumstances, the intestinal barrier can effectively prevent harmful substances, such as pathogens and endotoxins, from entering the bloodstream via the intestinal mucosa, thereby maintaining homeostasis (2). However, under conditions of stress, such as severe infections, surgical trauma, and ischemia-reperfusion injury, the intestinal barrier may be compromised, leading to intestinal barrier dysfunction (IBDF) (3). Patients are more likely to experience IBDF after major abdominal surgery (4). Research (5) has shown that intestinal infections caused by IBDF are an important cause of systemic inflammatory response syndrome, multiple organ failure, and even death. Therefore, early identification of high-risk patients and implementation of effective preventive measures are crucial to reduce the incidence of IBDF and improve treatment outcomes and quality of life. However, due to the lack of effective prediction and assessment tools, the early identification of IBDF remains challenging.

In previous studies (6, 7), biomarkers such as intestinal fatty acid binding protein (IFABP), D-lactate, and bacterial endotoxins have been widely used to evaluate IBDF. However, these indicators typically increase only after intestinal barrier damage occurs, meaning their changes often lag behind the actual pathological process (8, 9). Thus, although these markers can be used to monitor previously established IBDF, they cannot predict its occurrence, thus limiting the possibility of early identification and intervention. In recent years, research on the factors influencing the IBDF has gained considerable attention. Although some studies (10–12) have found that factors such as hyperglycemia, malnutrition, and platelet activation may be associated with an increased risk of IBDF, these studies mainly focused on exploring the role of a single factor and lack a comprehensive analysis of the interaction among multiple factors, resulting in limited predictive value for IBDF.

Currently, although risk prediction models based on clinical influencing factors have achieved satisfactory results in predicting other diseases, research on risk prediction models for IBDF remains relatively scarce. Given the serious harm that IBDF may cause and the current lack of research, it is necessary to develop a simple, accurate, convenient, and cost-effective prediction model to identify high-risk patients with IBDF in a timely manner.

In this study, we aimed to explore the perioperative clinical factors influencing IBDF and construct and validate a practical IBDF-nomogram model for personalized prediction of the risk of IBDF in patients after major abdominal surgery. The goal of this study is to effectively distinguish between low- and high-risk populations, thereby providing strong support for early personalized interventions for high-risk patients.

## Materials and methods

This study was approved by the Ethics Committee of the Second Affiliated Hospital of Kunming Medical University (Approval No. PJ-2024-124, 2024-01-05), and informed consent was obtained from all patients. This prospective observational study has also been registered at ClinicalTrials.gov (Registration No. NCT06596070). The conduct and reporting of the study comply with the Strengthening the Reporting of Cohort Studies in Surgery (STROCSS) guidelines (13).

### Study design and patient cohort

This prospective study included patients undergoing major abdominal surgery (defined (14) as an intraperitoneal operation with no primary involvement of the thorax, involving either luminal resection and/or resection of a solid organ associated with the gastrointestinal tract) at the Second Affiliated Hospital of Kunming Medical University and Kunming Haikou Hospital between January 2024 and January 2025. The inclusion criteria were as follows: (1) age >18 years; (2) scheduled for major abdominal surgery (radical surgery for gastric cancer, colorectal cancer, or pancreaticoduodenectomy); (3) voluntary participation in this study and signed an informed consent form. The exclusion criteria were as follows: (1) preoperative treatment with neoadjuvant radiotherapy, targeted therapy, or immunotherapy; (2) inflammatory bowel disease (ulcerative colitis, Crohn’s disease), acute pancreatitis, or obstructive jaundice; (3) use of antibiotics, non-steroidal anti-inflammatory drugs, probiotics, or opioids within 2 weeks; (4) severe heart, kidney, and liver dysfunction; (5) history of abdominal surgery within 3 months or preoperative acute bleeding and gastrointestinal perforation; and (6) incomplete clinical data. All participants were required to undergo bowel preparation the day before surgery. We calculated that the minimum sample size required to develop this prediction model was 260, based on the sample size evaluation method proposed by Riley et al. (15). According to the inclusion and exclusion criteria, 684 patients were included in the analysis. Among them, 480 patients who underwent surgery at the Second Affiliated Hospital of Kunming Medical University were assigned to the training cohort, while 204 patients who underwent surgery at Kunming Haikou Hospital were assigned to the external validation cohort.

### Diagnostic criteria

This study refers to the diagnostic criteria for IBDF from published literature (6) as follows: (1) patients with a critical illness that may lead to IBDF; (2) On the basis of the primary disease, the patient exhibits symptoms such as abdominal pain, diarrhea, bloating, constipation, gastrointestinal bleeding, food intolerance, and signs such as weakened or absent bowel sounds; (3) patients with elevated plasma endotoxin levels; (4) increased intestinal permeability or intestinal hypoperfusion; (5) positive bacterial cultures from blood or ascitic fluid without other obvious infectious foci. Among these, criteria (1) + (2) are necessary for diagnosis, while a confirmed diagnosis requires either (1) + (2) + (5) or (1) + (2) + (3) + (4). In this study, we utilized (1) + (2) + (5) as the diagnostic basis for IBDF.

To address the limitations of traditional bacterial culture methods, such as their low sensitivity and long detection time, we optimized the microbial detection protocol. Previous studies (16, 17) have confirmed that Escherichia coli, as the most common translocating species in intestinal flora translocation, its load in peripheral blood increases significantly within 24 h after intestinal barrier damage; detection of E. coli counts in peripheral blood is the most convenient and effective method to assess intestinal flora translocation. In accordance with Kong et al.’s methodology (6), we implemented real-time quantitative polymerase chain reaction (qPCR) to quantify E. coli 16S rDNA copy numbers in peripheral blood samples collected 24 h postoperatively. This approach not only enhances sensitivity but also shortens the detection cycle (18). In this study, all IBDF diagnoses were performed at 24 h postoperatively.

### Data collection

We selected potential IBDF-influencing factors by reviewing relevant literature and consulting experts. Preoperative data included sex, age, body mass index, hypertension, diabetes, malnutrition risk, history of abdominal surgery, history of change in bowel habits, and preoperative obstruction. Intraoperative data included surgical method (laparoscopic vs. open surgery), operative time, blood loss volume, infusion volume, and whether blood transfusion was administered. The recorded postoperative data included albumin (ALB), C-reactive protein, interleukin-6 (IL-6), procalcitonin, white blood cell counts, neutrophil, lymphocyte, neutrophil-to-lymphocyte ratio (NLR), platelets, and platelet-to-lymphocyte ratio at 24 h after surgery. Whether patients were admitted to the intensive care unit after surgery and the use of opioids was recorded. At 24 h postoperatively, 5 mL of peripheral blood was collected from the patient using EDTA anticoagulant tubes. Within 2 h after collection, the samples were centrifuged at 3000 × *g* for 10 min (4°C) to separate the plasma layer, which was then aliquoted into sterile EP tubes and stored at −80°C for subsequent analysis. Genomic DNA was extracted using the QIAamp DNA Blood Mini Kit (QIAGEN, Hilden, Germany), strictly following the manufacturer’s instructions. The detection of Escherichia coli 16S rDNA was performed on the CFX96 Touch Real-Time PCR System (Bio-Rad, United States) with specific primers and TaqMan probes. A standard curve was established using serially diluted plasmid standards, and the 16S rDNA copy number (copies/mL) was calculated based on the regression equation. To ensure accuracy, all samples were independently tested in triplicate, with the geometric mean adopted as the final result.

Nutritional assessments were conducted using the Nutritional Risk Screening (NRS) 2002. The score ranges from 0 to 7, with a score of ≥3 indicating a risk of malnutrition and a score of <3 indicates no risk of malnutrition. Changes in bowel habits mainly include increased frequency of bowel movements (> 3 times/day) or decreased frequency (< 3 times/week), alternating constipation and diarrhea, and changes in stool characteristics (e.g., watery, black, or mucus stools). The presence of preoperative obstruction was determined by gastroenteroscopy, gastrointestinal contrast, and computed tomography. The BMI was calculated by dividing weight by the square of height (kg/m^2^).

### Statistical analysis

All statistical analyses were performed with SPSS (version: 27.0) and R 4.3.1 software. Categorical variables are expressed as counts (%), and inter-group comparisons were conducted with the χ^2^ test or Fisher’s exact test. For continuous variables, those that followed a normal distribution are expressed as the mean ± SD and were analyzed using independent samples *t*-test. Continuous variables that did not follow a normal distribution are expressed as the median (interquartile spacing), and data comparisons were performed using the Mann–Whitney U test. Variables with *P-*values < 0.05 in univariate logistic regression analysis were included in the multivariate logistic regression analysis to identify independent predictors of IBDF (*P* < 0.05). The variance inflation factor (VIF) and tolerance were calculated to assess the collinearity assumption, where a VIF value <10 and tolerance >0.1 indicated no significant collinearity. The visualized nomogram predictive model was developed using the “rms” package in R software and further extended into a web-based dynamic nomogram through integration of the DynNom and Shiny packages. The model’s discrimination was evaluated based on the area under the receiver operating characteristic (ROC) curve. The Hosmer–Lemeshow goodness-of-fit test was used to assess model calibration, with *P* > 0.05 indicating a good model fit. The bootstrap method (resampling the original data 1000 times) was used for internal validation of the nomogram model and calibration curve creation. Decision curve analysis (DCA) and clinical impact curve (CIC) were used to evaluate the clinical applicability of the model. *P* < 0.05 was considered statistically significant.

## Results

### Patient characteristics

**FIGURE 1:**
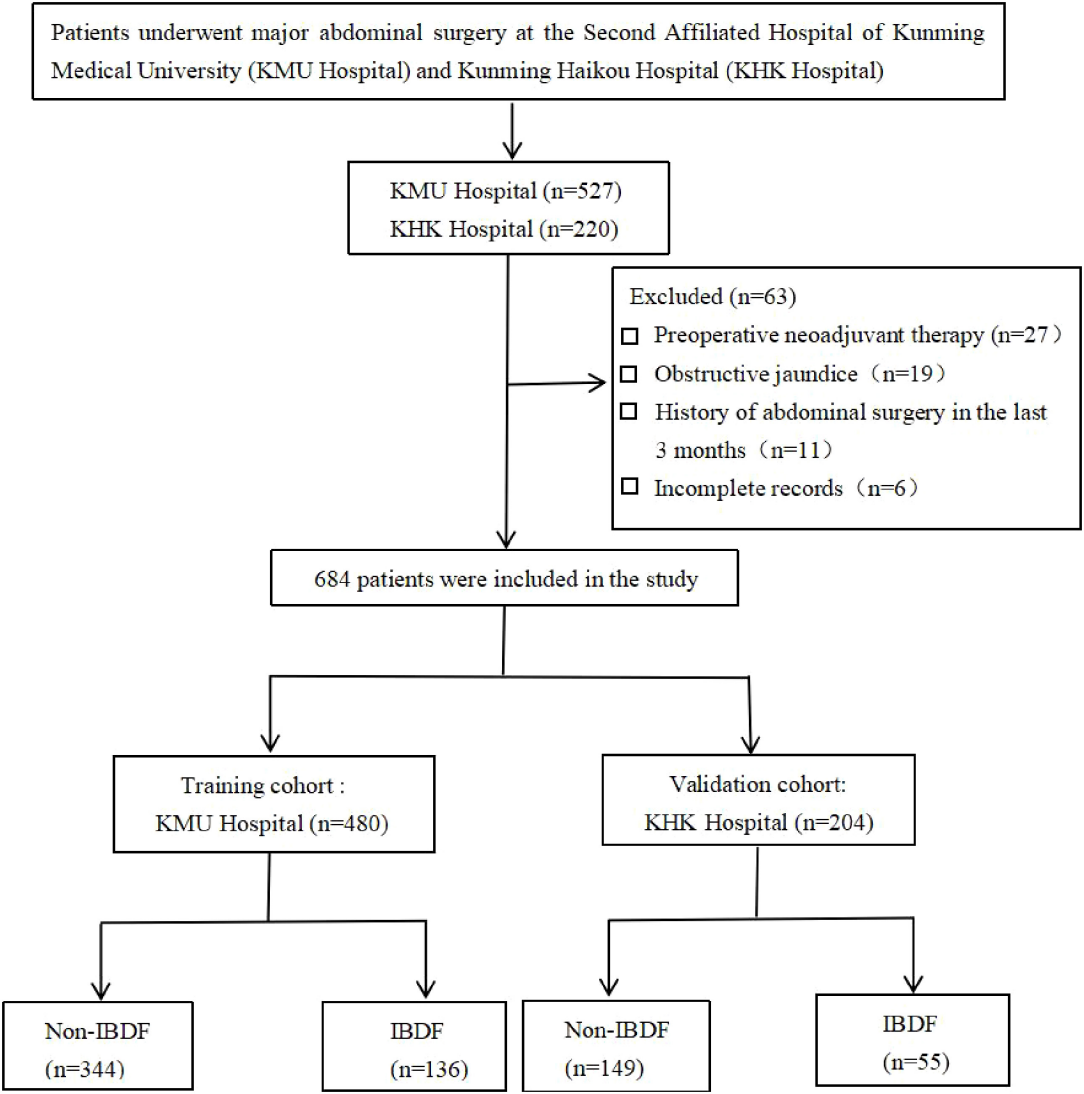
Flow chart for patient selection. IBDF, intestinal barrier dysfunction.

During the study period, 684 eligible patients were included in the analysis (*n* = 480, training cohort; *n* = 204, validation cohort). The flowchart of the study population selection is presented in Figure 1.

Table 1 lists the characteristics of patients in the training and validation cohorts. The training cohort included 268 males (55.8%) and 212 females (44.2%), with a median age of 62 years, while the validation cohort included 124 males (60.8%) and 80 females (39.2%), with a median age of 64.5 years. The training and validation cohorts had 136 (28.3%) and 55 (26.9%) patients with IBDF, respectively. The data from both cohorts were comparable, and the differences in various indicators were not statistically significant (*P* > 0.05).

**TABLE 1:** Baseline characteristics of the participants in the training and validation cohorts.

Characteristics	Total cohort (*n* = 684) M (Q25, Q75)/*N* (%)	Training cohort (*n* = 480) M (Q25, Q75)/*N* (%)	Validation cohort (*n* = 204) M (Q25, Q75)/*N* (%)	*P-*Value
Sex				0.266
Male	392 (57.3)	268 (55.8)	124 (60.8)	
Female	292 (42.7)	212 (44.2)	80 (39.2)	
Age, years	63.00 (54.00, 71.00)	62.00 (53.75, 71.00)	64.50 (55.00, 72.00)	0.244
Body mass index, kg/m^2^	22.51 (20.20, 24.90)	22.62 (20.20, 24.97)	22.46 (20.31, 24.62)	0.887
Diabetes				0.158
No	588 (86.0)	419 (87.3)	169 (82.8)	
Yes	96 (14.0)	61 (12.7)	35 (17.2)	
Hypertension				0.117
No	483 (70.6)	348 (72.5)	135 (66.2)	
Yes	201 (29.4)	132 (27.5)	69 (33.8)	
History of abdominal surgery				0.903
No	452 (66.1)	316 (65.8)	136 (66.7)	
Yes	232 (33.9)	164 (34.2)	68 (33.3)	
NRS 2002, points				0.470
<3	495 (72.4)	343 (71.5)	152 (74.5)	
≥3	189 (27.6)	137 (28.5)	52 (25.5)	
Change in bowel habits				0.365
No	425 (62.1)	304 (63.3)	121 (59.3)	
Yes	259 (37.9)	176 (36.7)	83 (40.7)	
Surgical method				0.910
Laparoscopy	590 (86.3)	415 (86.5)	175 (85.8)	
Open	94 (13.7)	65 (13.5)	29 (14.2)	
Operative time, h	3.80 (3.00, 5.00)	4.00 (3.00, 5.00)	3.60 (2.90, 4.60)	0.120
Blood loss, ml	100 (50, 200)	100 (50, 200)	100 (50, 200)	0.801
Infusion volume, ml	1800 (1300, 2500)	1800 (1350, 2500)	1750 (1200, 2362)	0.131
Opioids				0.733
No	404 (59.1)	281 (58.5)	123 (60.3)	
Yes	280 (40.9)	199 (41.5)	81 (39.7)	
ALB, g/L	30.75 (28.00, 33.90)	30.80 (28.00, 33.90)	30.60 (28.00, 33.75)	0.466
C-reactive protein, mg/L	55.29 (27.03, 99.81)	53.03 (26.20, 97.85)	60.91 (28.22, 106.71)	0.321
IL-6, pg/mL	52.80 (22.00, 127.25)	53.61 (22.23, 134.00)	51.32 (21.87, 101.20)	0.433
Procalcitonin, ng/mL	0.23 (0.10, 0.72)	0.23 (0.10, 0.73)	0.22 (0.11, 0.67)	0.964
White blood cell, 10^9^/L	8.64 (6.96, 10.84)	8.56 (6.87, 10.64)	8.78 (7.17, 11.46)	0.103
Neutrophil, 10^9^/L	7.24 (5.62, 9.31)	7.18 (5.53, 9.23)	7.36 (5.73, 9.75)	0.203
Lymphocyte, 10^9^/L	0.78 (0.53, 1.03)	0.75 (0.52, 1.02)	0.84 (0.56, 1.06)	0.059
NLR	9.85 (6.54, 14.47)	9.77 (6.36, 14.50)	9.91 (6.81, 14.36)	0.958
Platelets, 10^9^/L	189.00 (147.00, 235.25)	188.00 (146.00, 236.00)	189.50 (151.75, 235.00)	0.828
Platelet-to-lymphocyte ratio	249.74 (171.10, 348.72)	257.68 (172.86, 348.72)	235.32 (165.79, 347.91)	0.297
Preoperative obstruction				0.907
No	500 (73.1)	352 (73.3)	148 (72.5)	
Yes	184 (26.9)	128 (26.7)	56 (27.5)	
Intensive care unit				0.133
No	391 (57.2)	265 (55.2)	126 (61.8)	
Yes	293 (42.8)	215 (44.8)	78 (38.2)	
Blood transfusion				0.190
No	596 (87.1)	424 (88.3)	172 (84.3)	
Yes	88 (12.9)	56 (11.7)	32 (15.7)	

NRS2002, nutritional risk screening; ALB, albumin; IL-6, interleukin 6; NLR, neutrophil to lymphocyte ratio.

### Screening for predictive factors

To determine the factors influencing IBDF, we conducted univariate and multivariate logistic regression analyses of 26 variables in the training cohort (Table 2). The results showed that the surgical method (OR: 3.209, 95% CI: 1.075–9.578), operative time (OR: 1.586, 95% CI: 1.101–2.286), blood loss (OR: 1.003, 95% CI: 1.001–1.005), infusion volume (OR: 0.999, 95% CI: 0.998–1.000), IL-6 level (OR: 1.002, 95% CI: 1.001–1.004), NLR level (OR: 1.132, 95% CI: 1.020–1.255), ALB level (OR: 0.805, 95% CI: 0.716–0.905), and use of opioids (OR: 4.415, 95% CI: 1.214–16.059) were independent influencing factors of IBDF (*P* < 0.05).

**TABLE 2:** Univariate and multivariate analyses of factors associated with intestinal barrier dysfunction.

Characteristics	Univariate analysis		Multivariate analysis	
	OR (95% CI)	*P-*Value	OR (95% CI)	*P-*Value
Sex, male vs. female	0.682 (0.454–1.024)	0.065		
Age, years	1.033 (1.016–1.051)	<0.001	0.980 (0.949–1.012)	0.220
Body mass index, kg/m^2^	0.876 (0.822–0.932)	<0.001	0.961 (0.848–1.088)	0.529
Diabetes, yes vs. no	1.168 (0.652–2.091)	0.602		
Hypertension, yes vs. no	0.882 (0.562–1.385)	0.586		
History of abdominal surgery, yes vs. no	2.367 (1.571–3.565)	<0.001	2.147 (0.981–4.649)	0.053
NRS2002, ≥3 vs. <3 point	3.149 (2.061–4.811)	<0.001	0.994 (0.400–2.470)	0.990
Change in bowel habits, yes vs. no	2.281 (1.519–3.425)	<0.001	2.088 (0.959–4.547)	0.064
Surgical method, open vs. laparoscope	11.546 (6.262–21.289)	<0.001	3.209 (1.075–9.578)	0.037
Operative time, h	1.719 (1.486–1.989)	<0.001	1.586 (1.101–2.286)	0.013
Blood loss, ml	1.006 (1.004–1.008)	<0.001	1.003 (1.002–1.005)	0.040
Infusion volume, ml	1.001 (1.000–1.001)	<0.001	0.999 (0.998–1.000)	0.003
ALB, g/L	0.736 (0.688–0.788)	<0.001	0.805 (0.716–0.905)	<0.001
C-reactive protein, mg/L	1.020 (1.016–1.025)	<0.001	1.004 (0.995–1.012)	0.397
Procalcitonin, ng/mL	2.100 (1.651–2.670)	<0.001	1.223 (1.012–1.496)	0.052
IL-6, pg/mL	1.006 (1.004–1.007)	<0.001	1.002 (1.001–1.004)	0.017
White blood cell, 10^9^/L	1.096 (1.037–1.159)	<0.001	1.063 (0.924–1.222)	0.394
Neutrophil, 10^9^/L	1.197 (1.117–1.282)	<0.001	1.087 (0.855–1.383)	0.496
Lymphocyte, 10^9^/L	0.067 (0.031–0.146)	<0.001	1.595 (0.206–12.340)	0.655
NLR	1.193 (1.146–1.242)	<0.001	1.132 (1.020–1.255)	0.019
Platelets, 10^9^/L	0.994 (0.991–0.997)	<0.001	0.994 (0.985–1.003)	0.188
Platelet-to-lymphocyte ratio	1.003 (1.002–1.005)	<0.001	1.002 (0.997–1.008)	0.342
Opioids, yes vs. no	9.536 (5.960–15.258)	<0.001	4.415 (1.214–16.059)	0.024
Preoperative obstruction, yes vs. no	7.113 (4.540–11.144)	<0.001	2.073 (0.892–4.822)	0.090
Intensive care unit, yes vs. no	7.618 (4.787–12.123)	<0.001	1.134 (0.271–4.742)	0.864
Blood transfusion, yes vs. no	4.879 (2.730–8.720)	<0.001	0.736 (0.223–2.423)	0.614

NRS2002, nutritional risk screening; ALB, albumin; IL-6, interleukin 6; NLR, neutrophil to lymphocyte ratio; OR, odds ratio; 95% CI, 95% confidence intervals.

A multicollinearity test was conducted on the eight selected predictive factors, and the results showed that their tolerance was >0.1 and the VIFs were all <10, indicating that there was no multicollinearity among the eight variables.

### Risk prediction nomogram development

**FIGURE 2:**
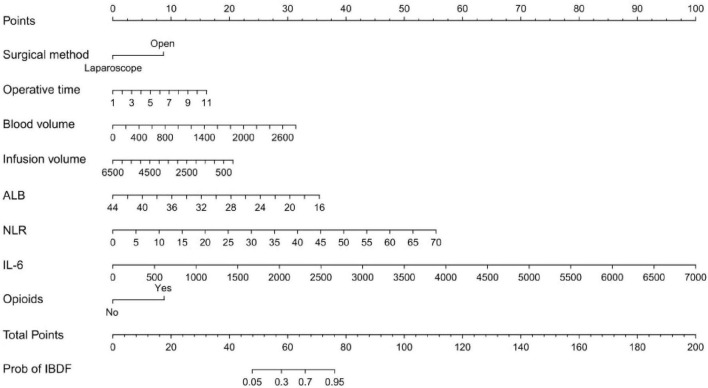
Nomogram for predicting the risk of intestinal barrier dysfunction in patients after major abdominal surgery. ALB, albumin; NLR, neutrophil-to-lymphocyte ratio; IL-6, interleukin-6.

**FIGURE 3:**
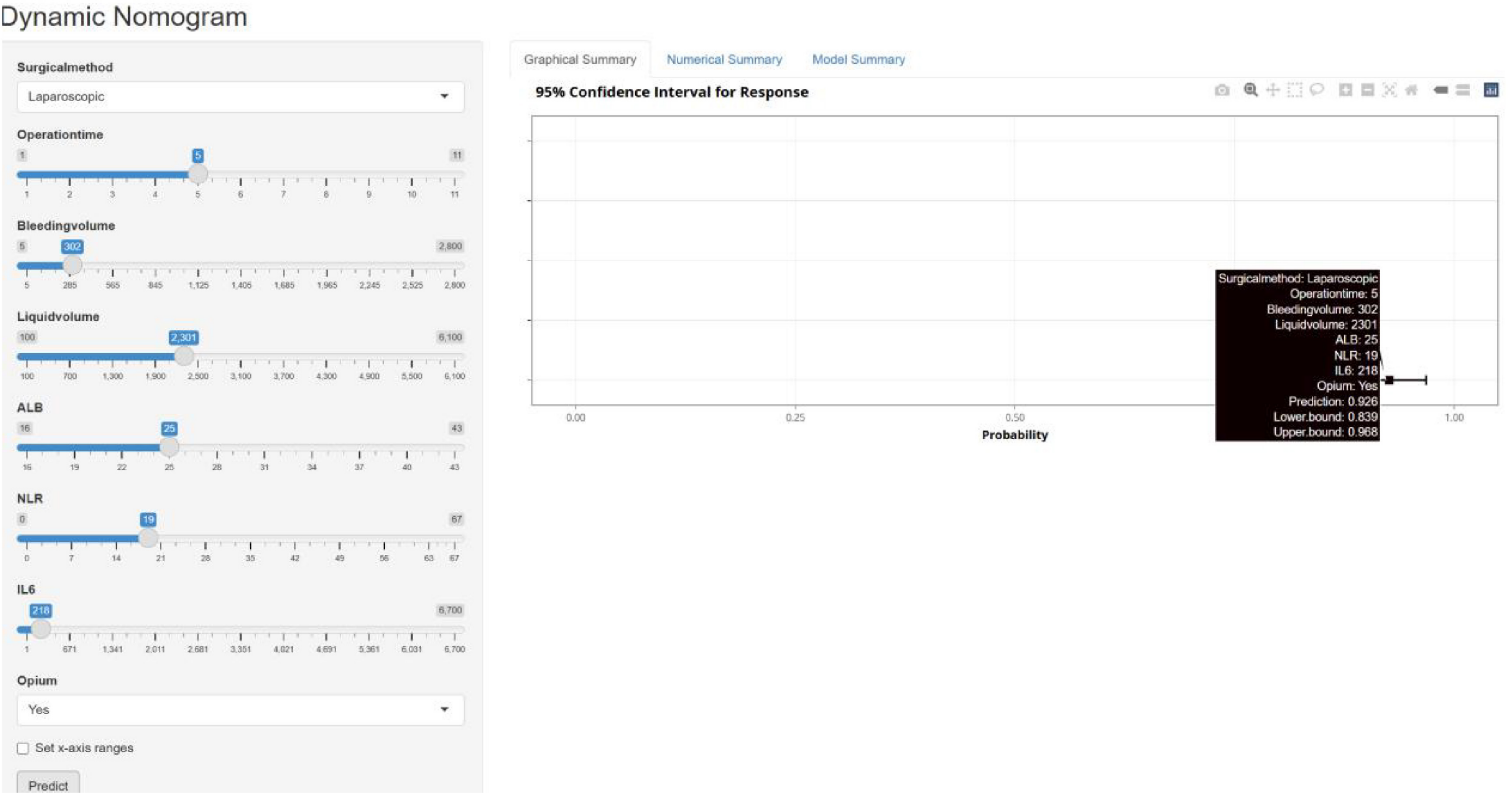
The web-based dynamic nomogram provides a real-time risk prediction interface with interactive parameter sliders and instantaneous probability updates.

Based on the eight selected independent predictive factors, we constructed an IBDF-nomogram model to predict the probability of IBDF occurrence in patients after major abdominal surgery (Figure 2). Each variable’s value was assigned a corresponding score, and the total score was obtained by summing the scores of individual variables. A vertical line was drawn down from the total score to estimate the probability of IBDF occurrence. The higher the probability, the higher the risk of IBDF. We also designed an online dynamic IBDF-nomogram^1^ that allows clinicians to quickly access the patient’s risk assessment results by adjusting the values of the predictor variables on their phones or computers (Figure 3).

### Model evaluation and validation

**FIGURE 4:**
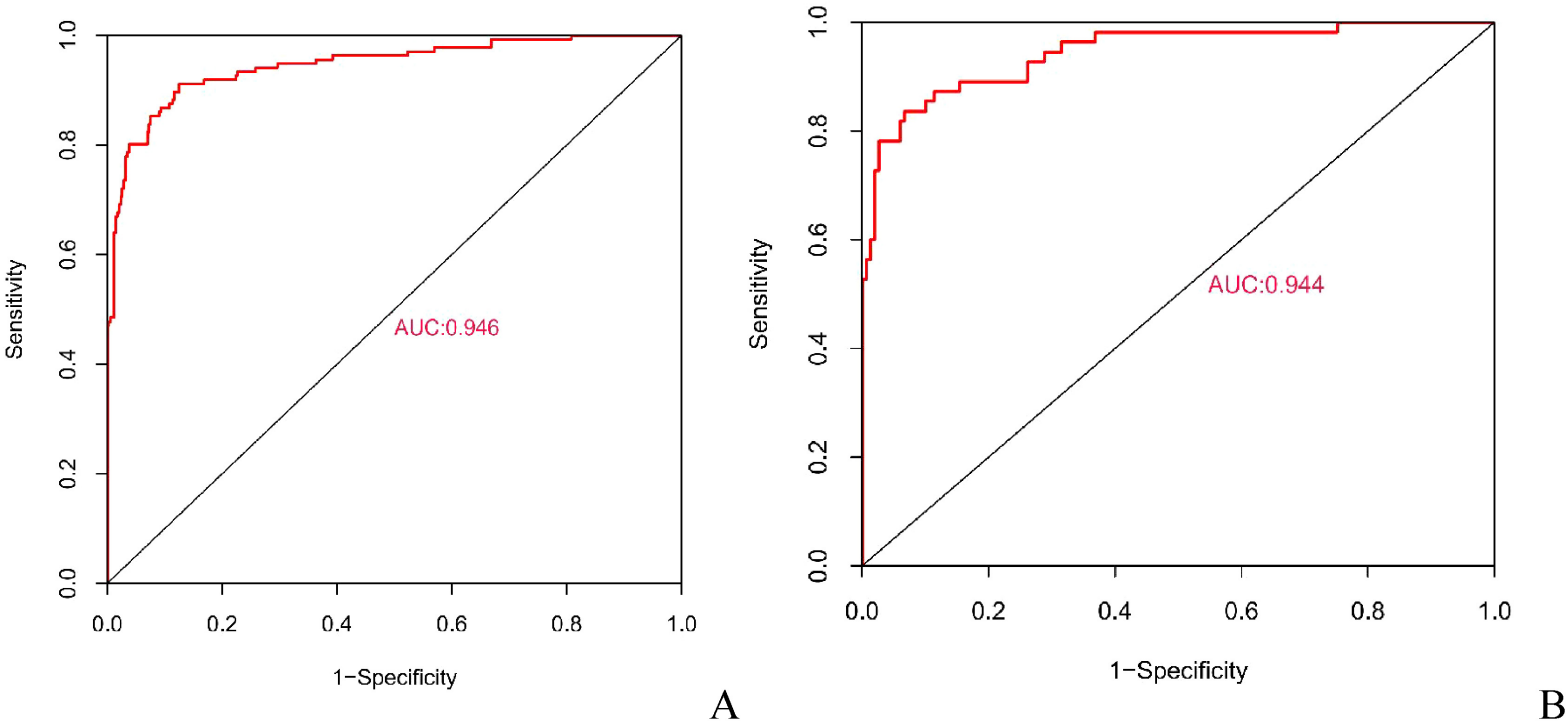
Receiver operating characteristic curves for evaluating the discriminative performance of the nomogram model in the training **(A)** and validation cohorts **(B)**. AUC, area under the curve.

**FIGURE 5:**
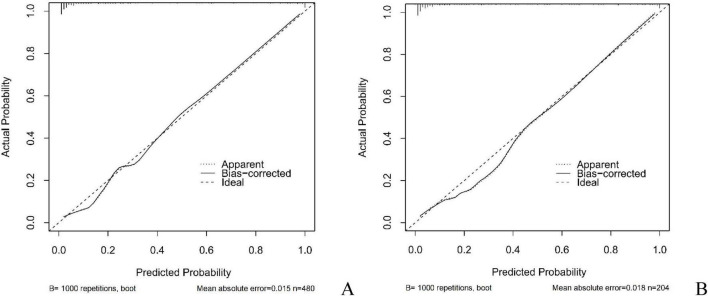
Calibration curves of the nomogram model. **(A)** Training cohort. **(B)** Validation cohort.

**FIGURE 6:**
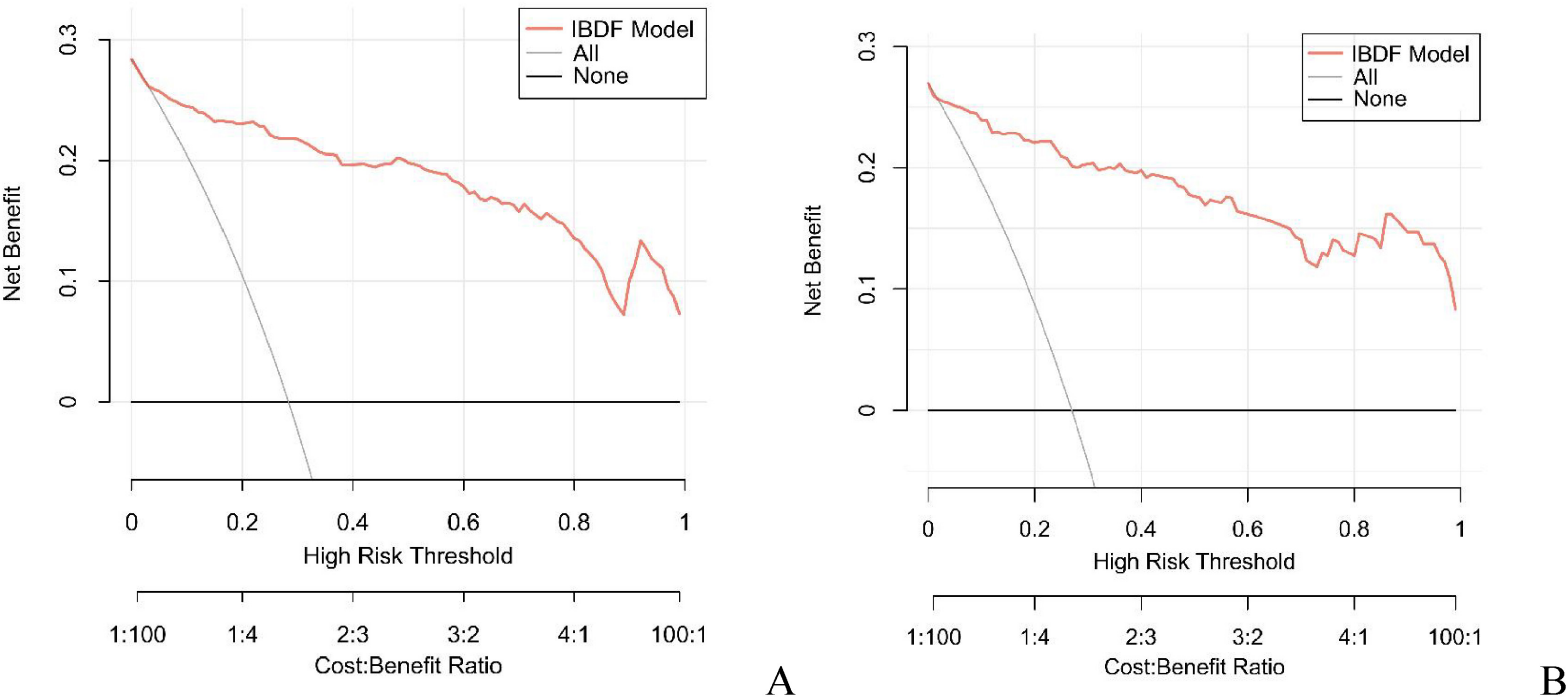
Decision curve analysis of the nomogram model in the training cohort **(A)** and validation cohort **(B)**. The horizontal axis represents the threshold probability, and the vertical axis represents the net profit. The black horizontal line indicates that no patients received intervention, while the gray curve indicates that all patients received intervention. The red curve corresponds to the risk nomogram. The generated curve showed that the net benefit of intervening based on the predicted values from the nomogram model was higher than that of no intervention or all intervention.

**FIGURE 7:**
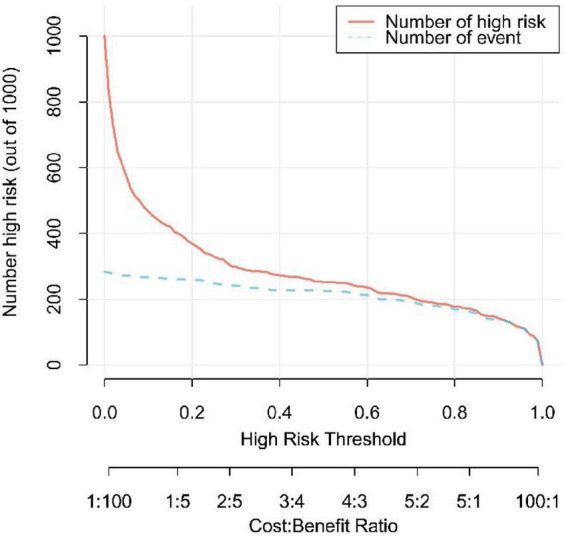
Clinical impact curve of the nomogram model. The red line represents the number of patients predicted by the model to be at high risk for IBDF, and the blue line showed the actual number of IBDF cases.

In this study, the AUC values of the nomogram model in the training and validation cohorts were 0.946 (95% CI: 0.921–0.970) and 0.944 (95% CI: 0.907–0.981), respectively, suggesting that the model has good discriminative ability in distinguishing whether patients develop IBDF (Figure 4). The calibration curve showed that the predicted probability of IBDF occurrence by the nomogram model was in good agreement with the actual outcomes. The *P-*values of the Hosmer-Lemeshow test for the training and validation cohorts were 0.720 and 0.716, respectively (Figure 5). We also plotted the DCA and CIC to assess the clinical application value of the predictive model. The DCA results indicate that when the probability thresholds for the training and validation cohorts exceed 0.03 and 0.02 respectively, using the nomogram model for treatment decisions yields a higher clinical net benefit compared to strategies of no intervention or intervention in all patients (Figure 6). The CIC results showed that the number of individuals identified as high risk by the model matches well with the number of those truly diagnosed with IBDF, indicating that the model had good clinical utility (Figure 7).

## Discussion

Intestinal barrier dysfunction (IBDF) may lead to systemic inflammatory response syndrome and multiple organ failure, severely jeopardizing patient health (5). However, there is currently a lack of effective tools to predict patients at risk of developing IBDF, which poses challenges for clinical prevention and management. In this study, we found that the surgical method, operative time, volume of blood loss, infusion volume, ALB level, IL-6 level, NLR level, and use of opioids were independent influencing factors for IBDF. The nomogram model constructed based on these factors can effectively predict the risk of IBDF among patients undergoing major abdominal surgery. The model demonstrated excellent discrimination and calibration abilities and good clinical practicality. In addition, the external validation results showed good accuracy and stability. This is the first model to use perioperative clinical factors to predict and evaluate IBDF and is available for free online. This model provides a basis for clinicians’ treatment decisions. Specifically, personalized preventive measures can be implemented for high-risk patients to reduce the risk of IBDF and avoid unnecessary treatment for low-risk patients, thereby achieving more precise preventive management and benefiting patients.

The surgical approach is an important factor influencing IBDF. George et al. (19) reported that, compared to laparoscopic surgery, open abdominal surgery significantly increases oxidative stress levels and intensifies intestinal barrier damage. Angenete et al. (20) found that in abdominal surgeries, the incidence of small intestinal obstruction after open surgery was at least four times higher than that after laparoscopic surgery. In our study, the risk of IBDF was 3.209-fold higher in patients undergoing open surgery than in those undergoing laparoscopic surgery. This may be because open surgery typically causes greater tissue trauma and stress responses, which may indirectly damage the integrity of intestinal epithelial cells, disrupt the structure of tight junction proteins, and thereby increase intestinal permeability (21–23). Additionally, operative time was found to be significantly correlated with intestinal barrier damage. Previous research (24) demonstrated that patients who underwent surgery for more than 4 h showed a significantly higher incidence of intestinal barrier damage than those who underwent surgery for less than 4 h (70.4% vs. 30.3%, *p* = 0.039). We also found that for each additional hour of operative time, the risk of developing IBDF increases by 1.586-fold (OR: 1.586, 95% CI: 1.101–2.286, *p* = 0.013). These findings suggest that operative time is an important influencing factor of IBDF; thus, shortening the operative time during clinical procedures may help decrease the incidence of IBDF.

We also found that excessive blood loss and insufficient fluid replacement can increase the risk of IBDF. Previous studies (25–27) have shown that excessive blood loss and insufficient fluid replacement can lead to decreased blood flow, activating the sympathetic nervous system and causing strong constriction of intestinal mucosal blood vessels, resulting in reduced tissue perfusion, intestinal mucosal ischemia and hypoxia. Tissue ischemia and hypoxia lead to oxygen delivery dysfunction and cellular energy depletion, which in turn triggers apoptosis and causes severe intestinal mucosal injury (28, 29). In addition, intestinal mucosal ischemia and hypoxia can stimulate the production of a large amount of inflammatory mediators, including tumor necrosis factor α, IL-6, and interleukin-1β (30). These inflammatory factors can disrupt tight junctions in the intestinal epithelial barrier, promoting intestinal epithelial barrier opening, mediated by myosin light chain kinase (MLCK), thereby increasing intestinal permeability and leading to IBDF (31, 32).

In the present study, patients with a higher NLR were more likely to develop IBDF. The increase in NLR may be due to an increase in neutrophil count, a decrease in lymphocyte count, or both (33). Research (34, 35) indicates that excessive neutrophil activation may lead to intestinal inflammation and impaired barrier function, whereas a reduction in lymphocytes may weaken immune function in the intestine, increasing the risk of intestinal barrier damage. Sikora et al. (36) reported that patients with impaired intestinal barrier function had significantly elevated NLR levels. Additionally, Halil et al. (37) showed that patients with high NLR levels had a significantly increased risk of intestinal ischemia and perforation. These studies support our findings. We believe that an elevated NLR indicates that patients are in a state of high inflammation and/or immune decline, thereby increasing the risk of IBDF.

ALB levels also influence the occurrence of IBDF. ALB is the protein component with the highest concentration in plasma, which not only maintains colloid osmotic pressure but also inhibits oxidative stress, maintains capillary integrity, and improves microcirculation (38, 39). Previous studies (40) have shown that hypoalbuminemia (serum albumin concentration <30 g/L) is significantly associated with increased intestinal permeability. Other studies (41, 42) have shown that lower ALB levels are an independent risk factor for pancreatic fistulas and gastrointestinal anastomotic fistulas in patients after abdominal surgery. Similarly, our findings suggest that low ALB levels are an independent risk factor for IBDF in patients after abdominal surgery.

The study also found that the use of opioids increases the risk of IBDF. Previous studies (43, 44) have indicated that opioids not only inhibit the movement of intestinal smooth muscle through opioid receptors such as μ-opioid and δ-opioid receptors, increasing the risk of intestinal dysfunction, but may also disrupt intestinal homeostasis by altering the composition of the gut microbiota. For example, opioid use has been shown to lead to a decrease in beneficial bacteria and an increase in pathogenic bacteria, thereby damaging the integrity of the gut epithelial barrier (45, 46). Additionally, research by Meng et al. (46) revealed that opioids inhibit the proliferation and repair capacity of intestinal epithelial cells, thereby weakening the barrier function of the intestine. The findings of these studies, along with our own, suggest that opioids disrupt intestinal barrier function and that clinicians should be cautious when using opioids during treatment.

The advantages of this study are as follows: we systematically explored the independent predictive factors of IBDF after major abdominal surgery for the first time and successfully constructed the IBDF-nomogram model, which provides an effective tool for the early identification of IBDF and fills the gaps in current research. Additionally, the predictors included in the model are all routine examination items during the patient’s hospitalization, and the data are easily accessible, which facilitates real-time monitoring of the patient’s condition. Moreover, to make the model more convenient to use, we developed an online dynamic IBDF-nomogram, allowing users to quickly obtain risk assessment results by simply adjusting the values of the predictive variables on their mobile phones or computers, greatly improving clinical decision-making efficiency. Overall, our model has strong clinical relevance and has the qualities of being non-invasive, convenient, quick, easy to access, high accuracy, and low cost, making it well-suited for practical applications in clinical settings.

However, this study also has certain limitations. First, due to the limited number of cases, we failed to include all types of patients undergoing abdominal surgery for analysis, which may have led to biased results. To enhance the generalizability of research findings, future studies should expand the sample size to include different types of patients undergoing major abdominal surgery and consider individual differences. Second, although this study systematically integrated critical perioperative variables (e.g., operative time, intraoperative blood loss), certain potential factors (e.g., American Society of Anesthesiologists Physical Status Classification System, surgical types) were not included in the analysis due to incomplete data or other constraints, which may influence the results. Finally, our model is currently externally validated using only a single medical center dataset. In future studies, we plan to conduct multicenter studies to expand the sample size and include populations from different geographic regions to further strengthen the reliability and generalizability of the model.

## Conclusion

In conclusion, to the best of our knowledge, this is the first study to develop and validate an IBDF-nomogram for predicting the risk of IBDF in patients after major abdominal surgery. This model may help clinicians identify high-risk patients with IBDF at an early stage and implement personalized prevention and management measures to improve patient prognosis. In the future, prospective multicenter, large-sample studies should be conducted to validate the effectiveness of this model.

## Data Availability

The original contributions presented in the study are included in the article/supplementary material, further inquiries can be directed to the corresponding author.

## Footnotes

1
https://emmazou.shinyapps.io/DynNomapp/
